# [Expression of Concern] Effects of cullin 4B on the proliferation and invasion of human gastric cancer cells

**DOI:** 10.3892/mmr.2025.13780

**Published:** 2025-12-17

**Authors:** Feng He, Xiu-Mei Cheng, Wen-Long Gu

Mol Med Rep 17: 4973–4980, 2018; DOI: 10.3892/mmr.2018.8509

Following the publication of this paper, it was drawn to the Editor's attention by a concerned reader that there was a possible duplication of cell-cycle analysis data shown in Fig. 4A on p. 4977. The authors were contacted by the Editorial Office to offer an explanation for this apparent anomaly in the presentation of the data in this paper; however, up to this time, no response from them has been forthcoming. Owing to the fact that the Editorial Office has been made aware of potential issues surrounding the scientific integrity of this paper, we are issuing an Expression of Concern to notify readers of this potential problem while the Editorial Office continues to investigate this matter further.

